# Hyperemesis gravidarum avec troubles ioniques sévères: à propos d'un cas

**DOI:** 10.11604/pamj.2015.20.264.6298

**Published:** 2015-03-19

**Authors:** Anouar Jarraya, Sahar Elleuch, Jawhar Zouari, Khaled Trigui, Abidi Sofiene, Mohamed Smaoui, Kamel Kolsi

**Affiliations:** 1 Service d'anesthésie réanimation, Hôpital Hedi Chaker, Sfax, Tunisie; 2 Service d'Anesthésie Réanimation, Hôpital Kremlin-Bicêtre, France; 3 Service de Gynécologie Obstétrique, Hôpital Hedi Chaker, Sfax, Tunisie

**Keywords:** Hyperemesis gravidarum, troubles ioniques, grossesse, Hyperemesis gravidarum, electrolyte disorders, pregnancy

## Abstract

L'hyperemesis gravidarum s'accompagne habituellement d'une perte de poids, d'une acétonurie et de troubles hydro-électrolytiques comme il peut également s'accompagner d'anomalies du bilan hépatique. Nous rapportons un cas de vomissements gravidiques à 10 semaines d'aménorrhée non traité et vu tardivement avec des troubles ioniques sévères associés à des répercussions cliniques dans un contexte de cytolyse, de cholestase et d'insuffisance rénale aigue. Ce cas a bien répondu au traitement médical.

## Introduction

Les nausées et les vomissements sont assez fréquents au premier trimestre de la grossesse et sont généralement bénignes. Parfois, ces symptômes sont exagérés ou prolongés définissant l'hyperemesis gravidarum qui se caractérise par plus de 5 épisodes de vomissements par jour avec une perte de poids de plus de 5% associés à des troubles hydro électrolytiques et à une acétonurie. Dans cet article nous rapportons un cas d'hyperemesis gravidarum menaçant le pronostic vital d'une jeune primigeste. Ce tableau s'est compliqué par des troubles ioniques très sévères associés à une insuffisance rénale aigue, à une cytolyse et à une cholestase importante vu que les vomissements ne sont pas traités depuis un mois.

## Patient et observation

La patiente a été en oligurie initialement et la bandelette urinaire a montré une acétonurie de 3 croix sans glycosurie et la glycémie au doigt a montré une glycémie à 1.05 g/l. A l’électrocardiogramme, on a constaté un élargissement du QRS avec onde U tout en gardant un rythme régulier à 122 bat/min et sinusal. La biologie faite en urgence a montré une hémoconcentration en faveur d'une déshydratation extracellulaire (hématocrite à 39.2%) et une hyponatrémie à 117 mmol/l en faveur d'une hyperhydratation intracellulaire associée à une hypokaliémie sévère à 1.38 mmol/l et une hypo chlorémie à 52 mmol/l. Le bilan hépatique a montré une cytolyse importante: ASAT à 396 UI et ALAT à 160 avec une cholestase: bilirubine directe à 73.2 et la bilirubine totale à 172.4, PAL à 345 sans insuffisance hépatique (TP à 82%). Une échographie abdominale a montré un foie augmenté de taille avec une flèche hépatique de 15.5 cm de contours réguliers et d’échostructure hyperéchogène homogène de surcharge métabolique: aspect évocateur de stéatose hépatique. La vésicule biliaire a été non distendue et à paroi fine avec absence de dilatation des vois biliaires intra-hépatique et de la voie biliaire principale. La patiente a eu un remplissage par 6 litres de sérum physiologique et 3 litre de Ringer lactate les 12 premières heures avec la supplémentation potassique sur une voie veineuse centrale à rythme de 1g/heure. Pour le traitement des vomissements on a prescrit l'oméprazole 40 mg /jour en perfusion avec du metoclopramide à la dose de 10 mg par 6 heures. Une perfusion d'albumine humaine a été prescrite devant une hypoalbuminémie à 45 g/l.

L’évolution était favorable avec amélioration clinique des vomissements et de la diurèse (50 ml/heure) et biologique avec une kaliémie à 2.58 mmol/l, une natrémie à 129 mmol/l et amélioration de la fonction rénale (créatinine à…µmol/l.). Le bilan hépatique a gardé la cholestase et la cytolyse à H12 d’évolution. Ensuite, on passé au sérum glucosé 5% standard à rythme de 2 litres par jour tout en gardant la suppléméntation potassique et le reste du traitement. A J3 d’évolution on a noté la correction de la kaliémie à 4.25 et disparition de la cytolyse ASAT à 65 et ALAT à 137 avec persistance de la cholestase (Bil.T= 103.8 et Bil.D= 42.3) d'où on a arrété la supplémentation potassique et l'alimentation entérale a été entamé avec succès par fractionnement des repas et la patiente à été mise sortante le lendemain.

## Discussion

Les nausées et les vomissements peuvent accompagner 50 à 90% des grossesses [1] avec un pic de fréquence à 9 SA. Ces signes disparaissent le plus souvent après 20 SA comme ils peuvent persister jusqu'au moment de l'accouchement. Ces symptômes sont plus fréquents chez la primigeste [1]. Dans quelques rares cas soit 0.3% à 1.5%, [2, 3] on peut assister à des formes prolongés et sévères de vomissements définissant alors l'hyperemesis gravidarum (HG) qui peut mettre en jeu le pronostic vital de la patiente comme le cas de notre patiente. L’étiologie reste encore mal connue. Il parait que les facteurs hormonaux sont les plus incriminés comme le ß HCG [4] et l'hyperthyroïdisme, à coté du facteur psychologique [5] surtout que 60% des HG développent une dépression secondaire [6] mais aussi l'infection chronique à Helicobacter pylori [7].

Les signes cliniques sont généralement non spécifiques et il est important d'exclure les maladies pouvant s'accompagner de nausées et de vomissements même en dehors de grossesse [8]. Ces maladies peuvent être des maladies gastro-intestinales tel que une appendicite, hépatite, pancréatite, iléus paralytique, cholécystite ou un ulcère gastroduodénal ou une gastroentérite comme elles peuvent être d'origine métabolique comme la maladie d'Addison ou la thyrotoxicose ou une décompensation diabétique. En effet, il existe des causes neurologiques comme la migraine ou les troubles vestibulaires ou l'encéphalopathie de Wernicke en plus des causes urogénitales comme la lithiase urinaire ou la pyélonéphrite ou un syndrome urémique. Au cours de la grossesse les nausées peuvent se voire lors des grossesses multiples ou la pré éclampsie ou la stéatose hépatique gravidique mais l'apparition récente des vomissement au terme de 10SA et la fréquence de ces signes dépassant les 5 fois par jour avec un amaigrissement et une acétonurie est plutôt en faveur de HG. Dans quelques cas rares mais graves on peut voire un ralentissement idéo moteur pouvant aller au délire et au coma comme on peut voire un jaunisse.

Les examens biologiques à demander doivent comporter l'hématocrite, l'ionogramme, les transaminases, la bilirubine et un bilan thyroïdien en cas de suspicion d'une thyrotoxicose et un bilan urinaire surtout l'acétonurie. Il faut noter que l'HG peut donner des perturbations du bilan hépatique surtout une cytolyse mais ne dépassant pas 4 fois la normale [2] selon l’étude de Morali et qui pourrait atteindre 10 à 20 fois la normale selon wallstedt [9]. Dans le travail de Morali et al., la corrélation entre les anomalies enzymatiques hépatiques et la cétonurie était faible, ce qui suggère que d′autres facteurs que le jeûne en sont responsables. Trois facteurs pourraient favoriser les modifications des tests hépatiques: la déshydratation, l′hyperoestrogénie et l′hyperthyroïdie. Des anomalies thyroïdiennes (thyroxine libre élevée ou thyroid stimulating hormone (TSH) basse) sont associées à l′hyperemesis gravidarum dans 50% des cas. La dysthyroïdie est certainement secondaire à l′hyperemesis gravidarum, comme d′ailleurs les anomalies hépatiques. Aussi bien les anomalies des tests hépatiques que l′hyperthyroïdie disparaissent avec le traitement de l′hyperemesis gravidarum. La créatinine est intéressante à la recherche d'une insuffisance rénale associée.

L’échographie abdominale et obstétricale est primordiale car elle permet d'exclure une cause organique des vomissements mais sa répétition peut aggraver les signes d'origine psychosomatique [10] (c'est-à-dire la conversion d'un stress psychologique sous formes de signes somatiques) ce qui explique l'importance de la bonne relation entre médecin et patiente avant tout traitement médical [11]. La prise en charge initiale dépend de la sévérité du tableau clinique et de l'acétonurie qui impose l'hospitalisation si elle dépasse les deux croix à visée d'une réhydratation intraveineuse et un traitement antiémétique adéquat. L'hospitalisation de la patiente est aussi obligatoire si elle ne peut pas boire suffisamment à fin d’éviter la déshydratation qui pourrait entretenir les vomissements. Un régime alimentaire à base de jus de fruits diluée et de biscuits salés peut être utile surtout avec fractionnements des prises alimentaires tout en évitant les repas copieux et le stress [12]. Un régime hyper protidique a un effet bénéfique sur les vomissement plus qu'un régime equicalorique à base d'hydrate de carbone [13]. Ces règles hygiéno-diététiques sont parfois insuffisant à maitriser les vomissements d'où le recours à une faible dose d'antiémétique qui a montré une efficacité supérieure par rapport au placebo [8]. Ondansetron est parmi les traitements les plus recommandés avec peu d'effets indésirables [14]. D'autres recommandent du metoclopramide pour améliorer la vidange gastrique ainsi que l'administration de la vitamine B [15]. Les antiémétiques et antihistaminiques comme la meclizine, dimenhydrinate et diphenhydramine peuvent être utilisés. La mirtazapine est un antidépresseurs utile pour les vomissements résistants aux traitements habituels [16]. Certains ont utilisés les corticoides [17]: dans un travail prospectif comparant l′efficacité de la méthylprednisolone (Solumédrol^®^) à celle de la prométhazine (Phénergan^®^) [18], l’équipe de Los Anglos a montré que l'effet antiémétique était comparable en plus qu'aucun cas du groupe solumédrol n'a été ré hospitalisé alors que 5 patientes du groupes prométhazine ont été réhospitalisées et dont 3 ont bien répondu au traitement par la méthylprednisolone. Les auteurs conseillent d′utiliser les corticoïdes en deuxième intention, en cas d′échec des antiémétiques. En France, la prométhazine est déconseillée au cours du 1 er trimestre de la grossesse, et c′est habituellement le métoclopramide qui est utilisé en première intention. Pour d'autres, des faibles doses d'halopéridol ont un pouvoir antiémétique [19] ainsi que l'association de diazepam avec le traitement antiémétique réduisait le séjour en milieu hospitalier [20]. A coté du traitement médical, un bon encadrement psychologique de la patiente est obligatoire et améliore le succès de la prise en charge [5].

## Conclusion

Le syndrome d'HG est une situation rare d’étiopathogénie multifactorielle qui peut dans quelques cas mettre en jeu le pronostic vital de la patiente. Une prise en charge multimodale précoce est essentielle.
